# The Lay Press and the Medical Profession

**Published:** 1895-05

**Authors:** 


### The Lay Press and the Medical Profession.

The *Cleveland Leader* in most candid fashion thus punctures the bubble of newspaper opposition to medical-practice acts:

"The truth is, we are ashamed to confess that the reason why it is easy to purge and guard the bar, and impossible, apparently, to protect the medical profession from defilement, must be sought in the large advertising patronage given to newspapers by quacks. For selfish and unworthy considerations the more cowardly and avaricious portion of the press is always willing to help the cormorants who masquerade under the guise of physicians, defeat bills to drive them out of business or out of Ohio. If there were no money for base newspapers in the toleration of the ignorant and unscrupulous persons who prey upon the public as doctors, they would very soon be expelled."—*The Medical Age.*

